# Evaluation of Reactivity of Monoclonal Antibodies Against Omp25 of *Brucella* spp.

**DOI:** 10.3389/fcimb.2020.00145

**Published:** 2020-04-21

**Authors:** Xin Yang, Zuoping He, Guoxia Zhang, Jinhui Lu, Hui Zhang, Hui Ren, Yanjun Tian, Heng Yang, Chuangfu Chen, Linhai Li, Yongshui Fu, Jean-Pierre Allain, Chengyao Li, Wenjing Wang

**Affiliations:** ^1^Department of Transfusion Medicine, School of Laboratory Medicine and Biotechnology, Southern Medical University, Guangzhou, China; ^2^The Fifth Affiliated Hospital of Sun Yat-sen University, Zhuhai, China; ^3^Department of Infectious Diseases, General Hospital of Heilongjiang Agricultural Reclamation Bureau, Harbin, China; ^4^School of Animal Science, Shihezi University, Shihezi, China; ^5^Department of Blood Transfusion, Zhujiang Hospital, Southern Medical University, Guangzhou, China; ^6^Department of Laboratory Medicine, General Hospital of Southern Theatre Command of PLA, Guangzhou, China; ^7^Guangzhou Blood Center, Guangzhou, China; ^8^Emeritus Professor of Transfusion Medicine, University of Cambridge, Cambridge, United Kingdom

**Keywords:** brucellosis, *Brucella*, Omp25, mAb, flow cytometry assay, diagnosis

## Abstract

Brucellosis is a serious zoonosis occurring mainly in developing countries, and its diagnosis is largely dependent on serologic detection and bacterial culture. In this study, we developed the murine monoclonal antibodies (mAbs) against a conserved and major outer membrane protein 25 (Omp25) of *Brucella* species (*B*. spp.) for use in clinical diagnosis. The mAbs to Omp25 were produced by hybridoma technique, which were utilized for developing various immunoassays for detection of *Brucellae*, including Western blot (WB), enzyme-linked immunosorbent assay (ELISA), immunochemical staining (ICS), immunofluorescence staining (IFS), and flow cytometry assay (FCM). A number of five mAbs (2B10, 4A12, 4F10, 6C12, and 8F3) specific to Omp25 were selected, including 2 IgG1, 2 IgG2a, and 1 IgG2b. Among them, mAbs 6C12, 8F3, and 4A12 reacted highly with *B. melitensis* (M5-90), *B. abortus* (S19, 104M, and 2308), and *B. suis strain* (S2). No cross-reactivity with *Yersinia enterocolitica* O:9, *Salmonella* spp., and *Escherichia coli* was found. By mapping Omp25 epitopes, mAb 6C12 was found as reacting with a semi-conformational epitope, and mAbs 4A12 and 8F3 as recognizing a different linear epitope, respectively. The paired mAbs were tested for detecting *Brucella* species, suggesting that 8F3 was suitable for solid phase capture and 6C12 or 4A12 was suitable for conjugation with HRP for detection of *Brucella* Omp25 in ELISA. The FCM was established by mAb 6C12 for detecting intracellular *Brucellae*-infected peripheral blood mononuclear cells (PBMCs) from brucellosis patients. In conclusion, mAbs against Omp25 are precious reagents for detection of *Brucellae* in clinical samples with various immunoassays. mAb 6C12-based FCM could be potentially used for the monitoring of therapeutic efficacy for brucellosis in clinical practice.

## Introduction

Brucellosis is a severe zoonotic disease distributed worldwide, especially in the developing world (Wang et al., 2015). Sheep, goats, cattle, and pigs infected by *Brucella* species are the main sources of human brucellosis (Ducrotoy et al., 2018). In the past 10 years, human brucellosis has increased rapidly in China. There were 37,947 new cases of human brucellosis nationwide (morbidity 2.7318/100,000) in 2018 as reported by the Chinese CDC (http://www.nhc.gov.cn/jkj/s3578/201904/050427ff32704a5db64f4ae1f6d57c6c.shtml). New cases of human brucellosis were reported in non-endemic areas such as the Guangdong province (Chen et al., 2014), which is located in the south of China and far away from the high prevalence areas such as Inner Mongolia, Heilongjiang, Xinjiang, and Shanxi in the north or west of China (Wang et al., 2015).

At present, diagnosis of brucellosis mainly depends on serological methods used to detect antibodies against *Brucellae* in infected animals or humans (Araj, 2010). Diagnosing brucellosis by means of *Brucellae* cultures themselves can take at least 10 days. Once a *Brucella* infection becomes chronic, patients are likely to carry the bacteria for their whole lives. Anti-*Brucellae* methods are the most important approach for treatment of brucellosis. However, currently there are no rapid, simple, and quantitative methods for the evaluation of therapeutic efficacy of brucellosis during hospitalization. *Brucella* outer membrane proteins (Omps) are excellent candidates for the serologic diagnosis of *a Brucella* infection and potential antigens for recombinant subunit vaccines against brucellosis (Ahmed et al., 2015; Yousefi et al., 2016). Omp25 is one major Omps of the *Brucella* species and is considered to be closely related to virulence of *Brucellae* (Salhi et al., 2003; Goel and Bhatnagar, 2012). *Brucella* species without Omp25 survive for a shorter period of time than wild-type strains in mice (Edmonds et al., 2001, 2002). As a structural protein, Omp25 is highly conserved in various types and subtypes of *Brucellae* and induces a strong immune response (Cloeckaert et al., 2002; Goel et al., 2013; Ma et al., 2015). Therefore, it might be a useful diagnostic target for brucellosis. In a previous study, an antibody to Omp25 was used to identify rough *Brucella* isolates by means of a latex coagglutination test (Bowden et al., 1997). In this study, we aimed to generate novel monoclonal antibodies (mAbs) to Omp25 and to develop new immunoassays for diagnosis of brucellosis or evaluation of therapeutic efficacy of brucellosis in clinical practice.

## Materials and Methods

### Bacterial Strains

Inactivated *Brucella* strains, including *B. melitensis* (M5-90), *B. abortus* (S19, 104M and 2308), and *B. suis* (S2), were provided from Shihezi University, Xinjiang, China. *Yersinia enterocolitica* O:9*, Salmonella* spp*., and Escherichia coli* (ATCC 23922) were provided from the Department of Microbiology, Southern Medical University (SMU), Guangzhou, China.

### Recombinant *Brucella* Omps

Recombinant proteins of Omp31, Omp19, Omp16, and periplasmic protein 26 (BP26) were produced from *B. melitensis* strain in the laboratory (Qiu et al., 2012; He et al., 2016; Li et al., 2017). Omp25 gene (642 bp) from *B. melitensis* M5-90 genomic DNA was cloned into the pET30a expression vector (Zhang et al., 2014; Yousefi et al., 2016), and designated as pET30a-Omp25. Recombinant Omp25 (rOmp25) was expressed as an inclusion body in *Escherichia coli* (DH5α) by induction with 1 mM IPTG. The rOmp 25 extract was denatured by 8 M urea and purified by Ni-NTA Agarose (GE Healthcare, Milwaukee, Wisconsin, USA), and then refolded by dialysis against 50 mM Tris-HCl buffer containing a declining gradient urea from 6, 4, 2, to 0 M (Qiu et al., 2012; Yousefi et al., 2016). Sodium dodecyl sulfate–polyacrylamide gel electrophoresis (SDS-PAGE) was utilized to analyze rOmp25 (Yousefi et al., 2016). The purified soluble rOmp25 was used for mouse immunization and development of serologic tests.

### Mouse Immunization and Monoclonal Antibody Production

BALB/c mice were obtained from the Animal Experimental Center of Southern Medical University (SMU), Guangzhou, China. BALB/c mice were immunized with purified rOmp25. The hybridoma cells, secreting mAbs to rOmp25, were generated and selected according to a previously reported method (Qiu et al., 2012; Patra et al., 2014; Li et al., 2017). To prepare the ascitis fluid, BALB/c mice sensitized by the liquid paraffin were injected subcutaneously with 10^6^/ml hybridoma cells. The mouse ascites fluid was collected and purified by Recombinant Protein G NUPharose Fast Flow, rProtein G NUPharose FF (Nuptec, Hangzhou, China) (Divya et al., 2018).

All animal experimentations were approved by Southern Medical University (SMU) Animal Care and Use Committee (permit numbers: NFYY-2008-043 and NFYY-2010-076). All mouse surgery was performed under anesthesia, and all efforts were made to minimize suffering of animals.

### mAb Isotyping

The isotype of mAbs was determined by IsoQuick Strips (a mouse mAb isotyping kit, Sigma-Aldrich, St Louis, Missouri, USA).

### Blood Samples From Patients and Blood Donors

Plasma, or peripheral blood mononuclear cells (PBMCs), were isolated from healthy blood donors in Guangzhou and Harbin blood centers. Blood samples from brucellosis patients were collected at the General Hospital of Agricultural Reclamation Bureau, Harbin, Heilongjiang, China. All blood donor or patient samples were confirmed for negativity or positivity to *Brucella* infection by Standard Agglutination Test (SAT) and Polymerase Chain Reaction (PCR) (Mohammadi and Golchin, 2018). The PBMCs were prepared according to manufacturer's instructions (Ficoll Pague PLUS, GE Healthcare Life Sciences, USA).

### Peptides

Peptides spanning 213 amino acids of Omp25 were synthesized as P1–P10 (Table S1) by a commercial company (Chinese Yuantai Company, Nanjing, China). A peptide of Omp31 (EP24: EYLYTDLGKRNLVDVD) was used as a negative control. The purity of all peptides was more than 90% of total weight.

### mAbs Titration and Affinity Measurement

The concentration of purified mAbs was determined by NanoDrop 2000 (Thermo Fisher Scientific, USA) as 1.14 mg/ml for 4F10, 2.1 mg/ml for 2B10, 7.4 mg/ml for 6C12, 3.8 mg/ml for 8F3, and 1.9 mg/ml for 4A12, respectively. The titer of mAbs was measured by indirect ELISA (Qiu et al., 2012; Divya et al., 2018). The Immuno MicroWell plate was coated with 5 μg/ml rOmp25 in 0.1 M carbonate buffer (CBS, pH 9.6) overnight at 4°C and blocked in 1% bovine serum albumin (BSA) for 1 h at 37°C. The purified mAbs as primary antibody were serially diluted from 1:100 to 1:1,968,300, and then added to microwell plate for 1 h at 37°C. ELISA plates were incubated for 1 h at 37°C with 1:10,000 diluted goat anti-mouse IgG and IgM HRP-conjugate antibodies (Rockland Immunochemicals, Inc, USA). The optical densities (OD) of reactions were detected with Epoch Microplate Spectophotometer (BioTek Instruments, Inc. Winooski, USA). mAb titer was finally defined as the dilution fold at OD_450_ value of 1 by indirect ELISA.

mAb's relative affinity was determined according to reactivity in different concentration of ammonium thiocyanate (NH_4_SCN) solutions ranging between 0 and 4 M/L for 30 min at room temperature (Pullen et al., 1986; Macdonald et al., 1988). The appropriate concentration of primary mAbs was determined on the titration curve, which corresponded to the optical density observed near the top of the curve's linear portion (Macdonald et al., 1988). All experiments were carried out in triplicate and the mean of OD_450_ values obtained from three independent experiments was used to calculate the relative affinity.

### Lentivirus-Mediated Omp25 Expression in Cells

The infectious recombinant lentivirus (LV-HAGE-Omp25) mediated Omp25 expression in 293FT cell as described previously, mimicking *Brucella* Omp25 antigen in the infected mammalian cells (Zhang et al., 2012; Li et al., 2017).

### Western Blot (WB)

The supernatant of sonicated proteins (SSPs) from *B. melitensis* (M5-90), *B. abortus* (S19, 104M, and 2308), and *B. suis* (S2) were considered as the native Omp25 lysates, which were prepared by breaking *Brucella* spp. stains with ultrasonication (Ultrasonic Apparatus XO-650, Xianou, Nanjing, China). The native membrane protein extracts (NMPs) was prepared by Membrane Protein Extraction Kit (Bestbio, China). The rOmp25 and native Omp25 were electrophoresed on SDS-PAGE and transferred to Polyvinylidene Fluoride membranes (PVDF membranes, Millipore, Billerica, Massachusetts, USA). The blotted membrane was incubated in 1:1,000 diluted mAb for 2 h at room temperature. The strip was washed in TBS containing 0.05% Tween®20 (TBST) and incubated with 1:5,000 dilution of goat anti-mouse IgG and IgM HRP-conjugate antibodies. The blot was visualized by adding immuno-chemiluminescence reagent (ECL, Millipore, Billerica, Massachusetts, USA). An HCV rNS3 mAb was used as unrelated negative control (Qiu et al., 2012). The SSPs from *E. coli* (ATCC23922) strain was used as negative control.

### Enzyme-Linked Immunosorbent Assay (ELISA)

In indirect ELISA, rOmp25 or native Omp25 was used as a coating antigen. Purified mAbs were used as the primary antibody at optimum working concentration. The microwell plate was incubated with a 1:10,000 dilution of goat anti-mouse IgG and IgM HRP-conjugate antibodies as reported previously (Tiwari et al., 2013; Ahmed et al., 2015). In peptide-ELISA, peptides P1–P10 were used to identify antigenic epitopes recognized by each mAb (Qiu et al., 2012; Li et al., 2017). The double-antibody sandwich ELISA (DAS-ELISA) was used to detect *Brucella* Omp25 by cross-matching mAbs in pair with a capture mAb coated on the microwell plate and a detection mAb conjugated with HRP (Luo et al., 2012).

### Immunofluorescent Staining (IFS)

293FT cells were transduced with recombinant lentivirus expressing Omp25 (LV-HAGE-Omp25) at multiplicities of infection (MOI = 5) for 8 h. The transduced 293FT cells were detected by immunofluorescent staining (IFS) with Omp25 mAbs as same as with Omp31 or BP26 described previously (Li et al., 2017). PBMCs were collected from two brucellosis patients (Guangzhou Center for Disease Prevention and Control) and were tested by IFS with mAb 6C12. PBMCs from a healthy blood donor were used as the negative control.

### Immunochemical Staining (ICS)

Intact *Brucella* were detected by using ICS (Li et al., 2017). Briefly, after pretreatment with 3% H_2_O_2_ and washing with PBS three times, the *Brucella* smears on the glass-slide reacted with Omp25 mAbs and saturated with goat anti-mouse IgG and IgM HRP conjugate (Rockland Immunochemicals, Inc, USA). Bacteria were visualized with diaminobenzidine (DAB) substrate for color development.

### Flow Cytometry Assay (FCM)

To explore the capacity of mAb to recognize intracellular Brucellae in PBMCs of patients with brucellosis, the purified mAb 6C12 was labeled with phycoerythrin (PE) by PE/R-Phycoerythrin Conjugation Kit according to the manufacturer's instructions (Abcam, Cambridge, United Kingdom). FITC-Anti-CD14 antibody (M5E2 clone; BD, Bioscience, USA) was used to recognize the lineage of mononuclear cells. PBMCs collected from 28 brucellosis patients and 55 non-Brucella-infected blood donors were stained for intracellular Brucellae by FACSCalibur flow cytometer with FITC-Anti-CD14 antibody (M5E2) and PE-6C12 specific to FCM Omp25 (Delaporte et al., 2008; Okumura et al., 2015). Briefly, PBMCs were treated with 5 μl FITC-M5E2 for 30 min at room temperature and cells were washed twice with sample buffer [PBS containing 1% bovine serum albumin (BSA; Sigma-Aldrich, St Louis, Missouri, USA)]. After fixation and permeabilization with Intracellular Fixation & Permeabilization Buffer (eBioscience, California, USA), PBMCs were incubated with PE-6C12 for 45 min at 4°C and washed three times. Finally, 300 μl of resuspended PBMCs were tested by FCM. All steps were performed in the dark. The data were analyzed by FlowJo software version v10.0. The cutoff for non-Brucella-infected PBMCs from healthy blood donors was established as 1% by FCM.

### Statistical Analysis

Computer software (SPSS, Version 20.0, SPSS, Inc., Chicago, IL) was used for statistical analysis. All experiments were repeated at least three times independently. The results were presented as the mean ± SD.

## Results

### Production and Identification of mAbs to *Brucella* Omp25

**Figure 1:**
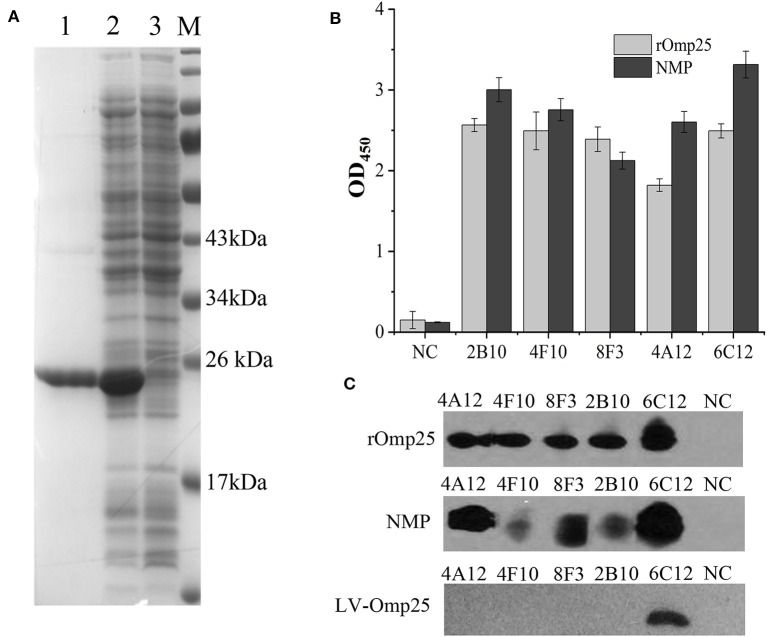
Purification and identification of recombinant Omp25. **(A)** The purified rOMP25 was analyzed by SDS-PAGE. Lane 1, purified Omp25 with 25 kD molecular weight; lane 2, cell lysate of pET30a-Omp25 transformed *E. coli* (DH5α) after IPTG induction; lane 3, cell lysate of pET30a-Omp25 transformed *E. coli* before IPTG induction. **(B)** Reactivity of five mAbs with rOMP25 or NMP of *B. melitensis* (M5-90 strain) in ELISA. **(C)** Reactivity of five mAbs with the denatured rOMP25 (1st panel) or NMP (2nd panel) of *B. melitensis* in WB. The third panel showed the denatured lentivirus expressed Omp25 in 293FT cells by WB. NC, negative control (recombinant NS3 to HCV).

Soluble rOmp25 was purified as an immunogen by Ni-NTA Agarose column. The purity of rOmp25 was about 95% of total proteins (Figure 1A). Five mAbs (2B10, 6C12, 8F3, 4A12, and 4F10) were selected for reacting with rOmp25 and NMPs of *B. melitensis* by ELISA, respectively (Figure 1B). Western blot analysis showed all five mAbs were highly reactive with denatured rOmp25, while among them three mAbs (6C12, 8F3, and 4A12) strongly reacted with the denatured NMPs of *B. melitensis* and two (2B10 and 4F10) reacted weakly (Figure 1C). In addition, only mAb 6C12 reacted with intracellular *Brucella* Omp25 expressed in 293FT cells (Figure 1C and Table 1). No mAbs reacted with other recombinant proteins of *Brucella* (Omp31, BP26, Omp19, and Omp16).

**Table 1:** Characterization of mAbs reactive to *Brucella* Omp25.

**mAb**	**Isotype**	**Titers**	**Relative** **affinity**	**Omp25** **WB**	**NMP** **WB**	**LV** **WB**	**Omp25** **ELISA**	**NMP** **ELISA**	**LV** **IFS**	***B. m*** **ICS**	**Epitope (aa)/peptide**
2B10	IgG2a(K)	1:2 × 10^4^	0.5–1.0	++	–	–	2.6	3.0	+	–	L(36–156)/P7
4A12	IgG1(K)	1:1 × 10^5^	1.0–1.5	++	++	–	1.8	2.6	+	+	L(24–42)/P2
4F10	IgG2b(K)	1:1 × 10^3^	0.5–1.0	++	–	–	2.4	2.7	+	–	L(24–42)/P2
6C12	IgG2a(K)	1:1 × 10^5^	1.0–1.5	++	++	+	2.5	3.3	++	+	SC(24–213)
8F3	IgG1(K)	1:1 × 10^5^	1.5–2.0	++	+	–	2.4	2.0	+	+	L(68–86)/P4

*NMP, native outer membrane proteins extracts of B. melitensis (B. m); LV, Lentivirus LV-HAGE-Omp25 transduced 293T cells; The numbers indicate the OD_450_ value in ELISA; The reactivity levels are indicated by ++ (strongly reactive), + (reactive), or – (non-reactive) by WB, IFS, and ICS; L, linear epitope; SC, semi-conformational epitope*.

### Classification and Affinity Titration of *Brucella* Omp25 mAbs

**Figure 2:**
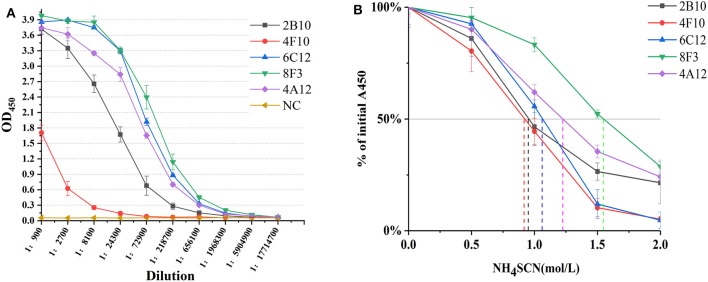
Determination of mAb titer and affinity. **(A)** Titration of mAb by an indirect ELISA. The mAb was serially diluted in 1:3. The optimum working concentration was determined for a midpoint of the steep portion of the curve. **(B)** The measurement of antibody relative affinity by thiocyanate elution assay. The affinity index was estimated by the molarity of NH_4_SCN causing 50% reduction from initial absorbance in the elution curves. All experiments were carried out in triplicate and the results were calculated from three independent experiments.

mAbs 2B10, 6C12, 8F3, 4A12, and 4F10 were identified as 2 IgG1, 2 IgG2a and 1 IgG2b, respectively (Table 1). Antibody titers were determined by ELISA at value of 1 at OD_450_. The titer of mAbs 6C12, 8F3, and 4A12 was measured up to 1:200,000, 2B10 up to 1:50,000, and 4F10 up to 1:1,000, respectively (Figure 2A). Correspondingly, the optimum working concentration of these five mAbs was 1.14 μg/ml (4F10), 0.042 μg/ml (2B10), 0.037 μg/ml (6C12), 0.019 μg/ml (8F3), and 0.01 μg/ml (4A12), respectively. An appropriate dilution of 1:2,700 for 2B10, 1:900 for 4F10, 1:8,100 for 6C12 and 8F3, and 1:2,700 for 4A12 was used as initial concentration of mAb to determine its relative affinity. The relative affinity of mAb was estimated by thiocyanate elution assay, calculated at 50% reduction from initial antibody reactivity reached by increasing molarity of NH_4_SCN in the elution curves (Figure 2B). Relative affinity of mAbs was around 1.0 (Table 1), among them the affinity of 2B10 and 4F10 ranged from 1.0 to 1.5, 4A12 and 6C12 from 1.0 to 1.5, and 8F3 from 1.5 to 2.0, respectively.

### Reactivity of mAbs With *Brucella* Species

**Figure 3:**
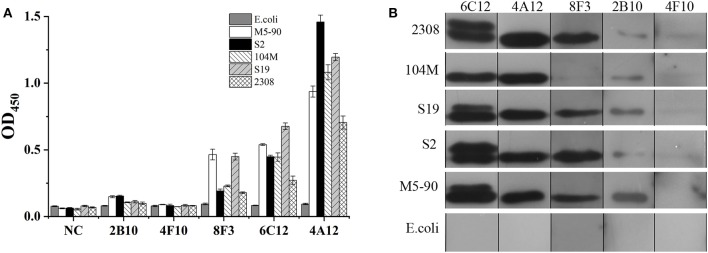
Detection of *Brucella* strains by mAbs to Omp25 in ELISA and WB. **(A)** The SSPs of M5-90, S19, 104M, 2308 and S2 strains were detected by an indirect ELISA. **(B)** Western blot analysis for identifying *Brucella* strains and non-*Brucella* stain with mAbs to Omp25. *Brucella* 2308 is a wild strain of *B. abortus*, 104M and S19 are vaccine strains of *B. abortus*, and S2 is a vaccine strain of *B. suis*, M5-90 is a vaccine strains of *B. melitensis*, respectively. *E. coli* (ATCC 23922) is used as a negative control.

The ability of mAbs to Omp25 from different strains of *Brucella*, the SSPs extracted from *B. melitensis* (M5-90), *B. abortus* (S19, 104M, and 2308), and *B. suis* (S2) were tested by ELISA and WB. mAbs 4A12, 6C12, and 8F3 reacted with all three of *Brucella* species by ELISA (Figure 3A). The reactivity of 4A12 and 6C12 reached a relatively higher level than 8F3, but 2B10 and 4F10 reacted at low level or did not react. Western blot analysis showed these mAbs had a reaction pattern similar to ELISA (Figure 3B). Specifically, mAb 6C12 presented two reactive bands with *Brucella* strains 2308 (*B. abortus*), S19 (*B. abortus*), S2 (*B. suis*), and M5-90 (*B. melitensis*), corresponding to Omp25 (the lower) and Omp25d (the upper), respectively. The other four mAbs reacted only with Omp25 (Figure 3B). No mAbs reacted with lysates of *Yersinia enterocolitica* O:9*, Salmonella* spp., and *Escherichia coli*.

### mAb Recognition for Omp25 Epitopes

**Figure 4:**
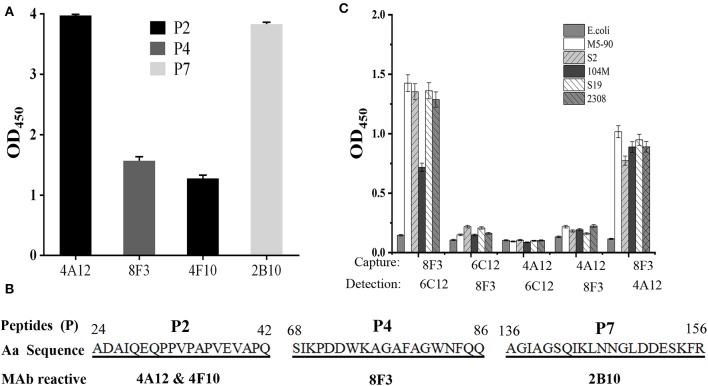
Epitope mapping of Omp25. **(A)** mAb reacted with peptides derived from Omp25 in ELISA. **(B)** mAb recognized linear epitopes within Omp25. Aa position is indicated at the beginning and the end of peptide sequence. mAb ID is indicated under the reactive peptide sequence. **(C)** Detection of *Brucellae* with cross-matching mAbs. The SSPs from *B. abortus* (S19, 104M, 2308), *B. suis* (S2) and *B. menlitensis* (M5-90) were used as antigens for cross-matching the mAb pair in DAS-ELISA. The capture mAb was used to coat microplates for Omp25 capture, and as detection mAb conjugated with HRP for detecting the captured Omp25 from various *Brucella* strains. *E. coli* was used as a negative control.

In order to identify the Omp25 antigenic epitopes, all mAbs were tested in ELISA with 10 peptides derived from the 213 amino acid (aa) sequence of *B. melitensis* Omp25. Four mAbs (4A12, 8F3, 4F10, and 2B10) reacted with three different peptides (Figure 4A) identified as recognition of linear epitopes. mAb 6C12 did not react with any peptides but with denatured native Omp25 (Figure 3), corresponding to the recognition of a semi-conformational epitope (Table 1). The amino acid sequences of the three linear epitopes of Omp25 are presented in Figure 4B (19–20 mers peptides P2, P4, and P7).

### Detection of *Brucellae* With Cross-Matching mAbs in ELISA

Five mAbs were tested for reactivity with Omp25 by cross-matching pairs with capture and detection antibodies by DAS-ELISA. Five pairs of mAbs (4A12/6C12-HRP, 4A12/8F3-HRP, 6C12/8F3-HRP, 8F3/4A12-HRP, and 8F3/6C12-HRP) reacted with rOmp25 (Table S2). These five pairs were tested for reactivity with the SSPs of 2308, 104M, S19, S2, and M5-90 strains from *B. melitensis, B. abortus*, or *B. suis*. Two mAb pairs, 8F3/6C12-HRP and 8F3/4A12-HRP, were found the be more reactive for detection of various *Brucella* species (Figure 4C).

### Detection of *Brucellae* by Immunostaining With Omp25 mAbs

To determine the ability of Omp25 mAbs to detect intracellular *Brucellae* or intact *Brucella* strains by microscopy, infected cells or intact bacteria were stained by IFS or ICS, respectively. mAbs 6C12, 2B10, and 4F10 showed a strong fluorescent reactivity with the lentivirus-Omp25 transduced 293FT cells by IFS (Figure S1A). In addition, by IFS with mAb 6C12 from two patients with brucellosis, visible fluorescent intracellular *Brucellae* infected PBMCs were observed, but not seen with controls from healthy blood donors (Figure S1B).

By ICS, the cultured *Brucella* could be seen by microscopy after staining with HRP-conjugated 6C12, 8F3, or 4A12 on a glass slide (Figure S2). The overall results are presented in Table 1.

### Clinical Detection of *Brucellae*-Infected PBMCs From Brucellosis Patients by FCM

**Figure 5:**
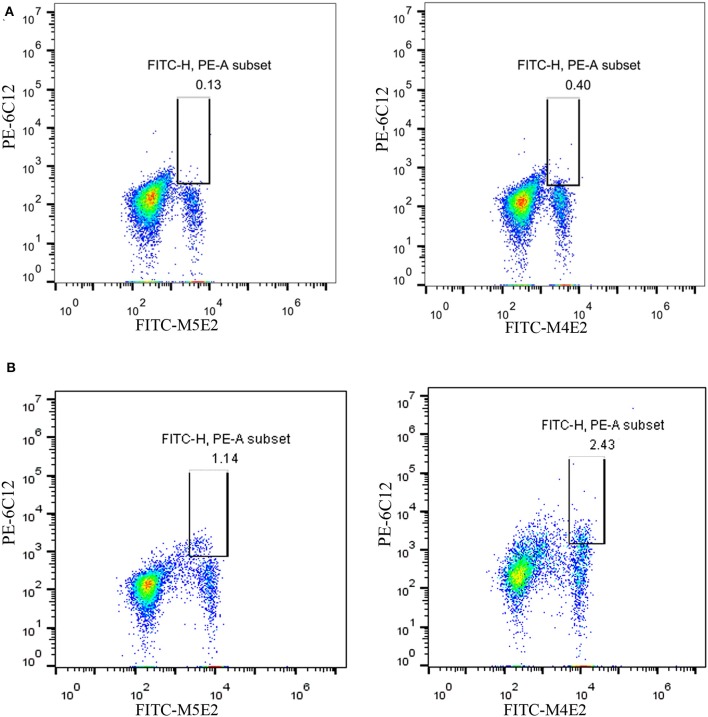
Detection of intracellular *Brucellae*-infected PBMCs from brucellosis patients by FCM. Monocytes were sorted by staining with mAb to CD14 (FITC-M5E2), and were further calculated by mAb to *Brucella* Omp25 (PE-6C12) as a percentage of total monocytes. **(A)** Detection of PBMCs from Healthy blood donors who were used as negative control. **(B)** Detection of PBMCs from brucellosis patients who were confirmed positive by SAT and PCR at administration to hospital.

On the basis of the above analysis, mAb 6C12 was considered the most functional antibody for *Brucella* detection. Hence, mAb 6C12 was selected for labeling with PE to detect intracellular *Brucellae*-infected PBMCs by FCM (Figure 5). The mononuclear cells were sorted from freshly isolated PBMCs by FITC-Anti-CD14 antibody (M5E2), and the ratio of *Brucella*-infected monocytes was calculated by staining with PE-6C12. Stained monocytes from healthy blood donor controls over brucellosis patients were stratified as the cutoff or threshold between two levels of intracellular *Brucellae*: <1.0%, indicating a lower level or negative intracellular *Brucellae*-infected cells (Figure 5A), and ≥1.0%, indicating a higher level of intracellular *Brucellae*-infected cells (Figure 5B). A total of 28 brucellosis patients and 20 blood donors were tested by FCM with both mAbs M5E2 and 6C12, showing that 46.4% (13/28) of brucellosis patients but none of blood donors carried intra-PBMCs *Brucellae* (≥1.0%) (Table 2). Only one patient (No. 8) had 1.13% of stained monocytes with unclear boundary by FCM. These results suggested that FCM with mAb 6C12 might be a practical assay to determine the frequency of intracellular *Brucellae*-infected PBMCs in individual brucellosis patients with clinical diagnosis.

**Table 2:** Characterization of blood samples and detection results of *Brucellae*-infected PBMCs by FCM with mAb PE-6C12.

**Number**	**Sample source**	**Status**	**FCM (%)**	**PCR**	**SAT**
1	Brucellosis patient	Acute	0.99	+	1:200
2	Brucellosis patient	Chronic	0.39	+	1:100
3	Brucellosis patient	Acute	1.59	+	1:100
4	Brucellosis patient	Chronic	0.57	+	1:100
5	Brucellosis patient	Chronic	0.97	+	1:400
6	Brucellosis patient	Chronic	0.61	+	1:100
7	Brucellosis patient	Acute	1.26	+	1:800
8	Brucellosis patient	Acute	1.13 (unclear boundary)	+	1:400
9	Brucellosis patient	Acute	2.04	+	1:50
10	Brucellosis patient	Acute	0.73	+	1:100
11	Brucellosis patient	Acute	0.79	+	1:100
12	Brucellosis patient	Chronic	2.41	+	1:50
13	Brucellosis patient	Acute	1.19	+	1:800
14	Brucellosis patient	Acute	0.69	+	1:400
15	Brucellosis patient	Chronic	0.75	+	1:100
16	Brucellosis patient	Chronic	0.8	+	1:200
17	Brucellosis patient	Acute	0.79	+	1:100
18	Brucellosis patient	Chronic	1.38	+	1:100
19	Brucellosis patient	Chronic	0.65	+	1:100
20	Brucellosis patient	Chronic	1.37	+	1:50
21	Brucellosis patient	Acute	1.18	+	1:100
22	Brucellosis patient	Acute	1.15	+	1:400
23	Brucellosis patient	Acute	1.97	+	1:200
24	Brucellosis patient	Chronic	2.43	+	1:50
25	Brucellosis patient	Chronic	3.12	+	1:100
26	Brucellosis patient	Chronic	4.14	+	1:50
27	Brucellosis patient	Acute	0.43	+	1:100
28	Brucellosis patient	Chronic	0.46	+	1:50
29	Blood donor		0.49	–	–
30	Blood donor		0.31	–	–
31	Blood donor		0.18	–	–
32	Blood donor		0.41	–	–
33	Blood donor		0.1	–	–
34	Blood donor		0.4	–	–
35	Blood donor		0.15	–	–
36	Blood donor		0.22	–	–
37	Blood donor		0.28	–	–
38	Blood donor		0.25	–	–
39	Blood donor		0.3	–	–
40	Blood donor		0.58	–	–
41	Blood donor		0.23	–	–
42	Blood donor		0.37	–	–
43	Blood donor		0.47	–	–
44	Blood donor		0.29	–	–
45	Blood donor		0.35	–	–
46	Blood donor		0.52	–	–
47	Blood donor		0.46	–	–
48	Blood donor		0.24	–	–

*FCM, flow cytometry; SAT, standard agglutination test*.

## Discussion

Several early studies focused on the immunogenicity and protective activity of *Brucella* Omp25 (Bowden et al., 1998). In this study, three non-overlapping linear epitopes (P2, P4, and P7) were recognized by mAbs 4A12 and 4F10, 8F3, and 2B10, respectively (Figure 4B). Previously mAb A59/05F01/C09 was reported to recognize a liner epitope of Omp25 (aa 24-43) (Salhi et al., 2003), which overlaps with the epitope P2 (aa24-42) recognized by mAbs 4A12 and 4F10 in this study. P1 peptide (aa1-30) has seven amino acids (24-ADAIQEQ-30) overlapping with P2 peptide (Table S1) but does not react with these mAbs, suggesting that mAbs 4A12 and 4F10 likely recognize the linear epitope PPVPAPVEVAPQ (aa 31-42) within Omp25. Another mAb A76/02C12/C11 to Omp25 was reported to strongly bind to rough *Brucella* isolates except *B. ovis* tested with a latex co-agglutination assay (Bowden et al., 1997), but the epitope was not clearly defined. In addition to the three different linear epitopes (P2, P4, and P7) revealed by mAbs 4A12 and 4F10, 8F3, and 2B10, a semi-conformational epitope within the denatured native *Brucella* Omp25 was identified by mAb 6C12 recognition. Interestingly, mAb 6C12 recognized two bands of 25 kD and 26 kD proteins from *Brucella* strains in WB (Figure 3B). The larger band (26 kD) was designated as Omp25d as previously described (Salhi et al., 2003), while the other four mAbs reacted only with the linear epitopes of 25 kD protein (Omp25). This might explain that 6C12 had better binding capacity in FCM than other mAbs for detecting *Brucellae* of PBMCs in comparison with mAb 5H3 to Omp31 or 5A5 to BP26 (Yang et al., 2019). These Omp25 mAbs had no cross-reactivity with *Yersinia enterocolitica* O:9*, Salmonella* spp., and *Escherichia coli* reported previously (Muñoz et al., 2015). However, previous studies found the high serological cross-reactivity between *Brucella* and other *Alphaproteobacteria* such as *Ochrobactrum* and *Sinorhizobium* (Cloeckaert et al., 1999; Velasco et al., 2000; Delpino et al., 2004). By aligning *Brucella* Omp25 aa sequence with *Ochrobactrum anthropi* and *Rhizobiales* 63-22 strains, the high homologous sequences are observed including the linear epitopes (P2, P4, and P7) within *Brucella* Omp25 recognized by mAbs 4A12/4F10, 8F3, and 2B10 (Figure S3), which indicate the cross-reactivity of these mAbs may exist in other *Alphaproteobacteria*.

*B. melitensis, B. abortus*, and *B. suis* are three major pathogenic strains for human brucellosis transmitted from infected sheep and goats, cattle, or pigs (Chen et al., 2014; Ducrotoy et al., 2018). Among five mAbs, 8F3, 4A12, and 6C12 had higher relative affinity (>1) reacted with Omp25 of five strains (M5-90, S19, 104M, 2308, and S2) from three *Brucella* in various immunoassays. mAb 8F3 presented a strong capacity as a capture antibody (Figure 4C), while 6C12 and 4A12 appeared to be the best detection antibody when conjugated with HRP or fluorescein (Figure 4C, Figures S1, S2). The best-matched antibody pairs were 8F3/6C12-HRP and 8F3/4A12-HRP for detection of *Brucellae* in DAS-ELISA, while mAb 6C12 was the most suitable for detection of intracellular *Brucellae* in PBMCs from patients by FCM (Figure 5).

Currently, there is no an optimal immunoassay for detecting intracellular *Brucellae* in terms of counting the infected monocytes for evaluation of anti-*Brucella* efficacy in clinical treatment of brucellosis. Conventional method of blood culture requires a biosafety level 3 laboratory (BSL-3) and takes 1–2 weeks (Pappas et al., 2006). In this study, we sought to develop an Omp25 mAb-based FCM assay to detect intracellular *Brucellae*-infected PBMCs from brucellosis patients, which might be helpful for evaluating therapeutic efficacy by determining the percentage of *Brucella*-infected monocytes before or after treatment of individual patients with brucellosis. By analyzing 28 patients with brucellosis, who were found positive upon admission to hospital by SAT and PCR, eight out of 28 (28.6%) and five out of 28 (17.8%) brucellosis patients had more than 1 or 2% intracellular *Brucellae* infected PBMCs, respectively. Overall, 46.4% (13 out of 28) patients carried a higher level (>1%) of intracellular *Brucellae* monocytes and 53.6% (15 out of 28) patients had a lower level (<1%) or no detectable intracellular *Brucellae* monocytes (Table 2). The ratios of mAb 6C12-stained monocytes from PBMCs were consistent with the amounts of intracellular *Brucellae* in brucellosis patients. In our previous study, we utilized IFS for detecting *Brucella-*infected PBMCs stained with Omp31 mAbs (Yang et al., 2019) and found 50–70% positive PBMCs in patients receiving zero to three treatment courses of anti-*Brucella* drugs. IFS is a qualitative test unsuitable to quantify the number of *Brucella*-infected cells in a patient's PBMCs. The selected mAb 6C12 has been shown effective for detection of intracellular *Brucella* by IFS (Figure S1). However, the established FCM with mAb 6C12 specific to Omp25 has an advantage over IFS for counting the frequency of intracellular *Brucellae*-infected monocytes and might be a potential assay for evaluation of therapeutic efficacy of brucellosis patients in clinical practice.

In conclusion, mAbs 8F3 was suitable as an immobilized capture antibody, and 6C12 or 4A12 was suitable as a conjugated detection antibody. These mAbs specific to *Brucella* Omp25 appear precious reagents in various immunoassays, such as WB, ELISA, ICS, IFS, and FCM assays for detection of different *Brucella* species in the diagnosis of brucellosis or evaluation of therapeutic efficacy of anti-*Brucella* treatment in clinical practice.

## Data Availability Statement

All datasets generated for this study are included in the article/Supplementary Material.

## Ethics Statement

The animal study and studies involving human participants were reviewed and approved by Southern Medical University (SMU) Nan Fang Hospital Medical Ethics Committee (permit numbers: NFYY-2009-23). The patients/participants provided their written informed consent to participate in this study.

## Author Contributions

WW, CL, and XY participated in the study design, analysis of data, and writing of the manuscript. XY, GZ, CC, LL, and YF collected blood samples and interpreted the patient data regarding the brucellosis disease. XY, ZH, JL, YT, HZ, HR, and HY performed the laboratory examination. J-PA analyzed the data and revised the manuscript. All authors read and approved the final version of manuscript.

## Conflict of Interest

The authors declare that the research was conducted in the absence of any commercial or financial relationships that could be construed as a potential conflict of interest.
