# Supplementary material for: Evaluation of Reactivity of Monoclonal Antibodies Against Omp25 of Brucella spp
Source: Front Cell Infect Microbiol. 2020 Apr 21;10:145. doi: 10.3389/fcimb.2020.00145 (PMC7187720; doi:10.3389/fcimb.2020.00145)
Supplement: Supplementary file 1 [file Data_Sheet_1.pdf]

Supplementary materials

TABLE S1

Peptide sequence of Omp25

**Peptide**	**Sequence**	**Position**
P1	TLKSLVIVSAALLPFSATAFAADAIQEQ	3-30
**P2**	ADAIQEQPPVPAPVEVAPQ	24-42
P3	YSWAGGYTGLYLGYGWNKAKTSTVG	43-67
**P4**	SIKPDDWKAGAFAGWNFQQ	68-86
P5	DQIVYGVEGDAGYSWAKKSKDGL	87-109
P6	EVKQGFEGSLRARVGYDLNPVMPYLT	110-135
**P7**	AGIAGSQIKLNNGLDDESKFR	136-156
P8	VGWTAGAGLEAKLTDNILGRV	157-177
P9	EYRYTQYGNKNYDLAG	178-193
P10	TTVRNKLDTQDFRVGIGYKF	194-213
Ep24 (Omp31)	EYLYTDLGKRNLVDVD	

Red characters mean the identical amino acids to other peptides.

Bold characters mean the peptide contains a linear epitope of Omp25 recognized by mAbs.

TABLE S2

Cross matching of HRP labeled mAbs to Omp25 in pair by DAS-ELISA

**Detection mAbs**	**Capture mAbs**				
	2B10	4F10	4A12	6C12	8F3
HRP-2B10	0.2	0.2	0.2	0.7	0.6
HRP-4F10	0.2	0.2	0.3	0.3	0.9
HRP-4A12	0.8	0.1	0.7	0.8	6.5
HRP-6C12	0.9	0.3	4.1	1.3	1.0
HRP-8F3	1.0	0.3	4.3	6.2	1.4

Reactivity was presented as S/CO. Cutoff (CO) was calculated as mean +3SD of OD_{450} values from negative control in ELISA.

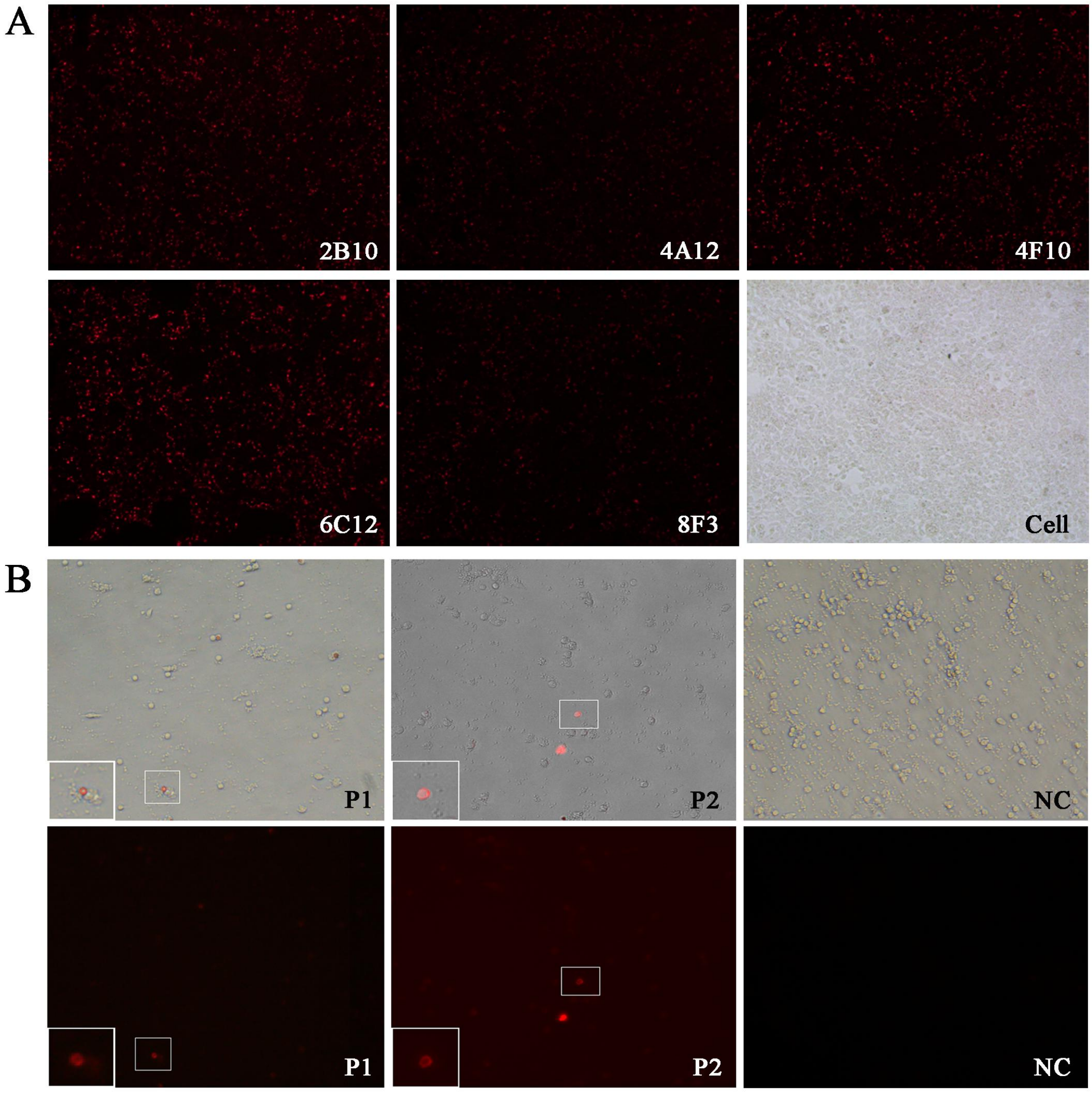

Figure S1. Staining of intracellular *Brucella* Omp25 in cells by IFS with mAbs, **(A)** Lentivirus LV-HAGE-Omp25 transduced 293FT cells were stained by IFS with mAbs specific to Omp25, respectively. 293FT cells were a control of negative cells observed in white light. **(B)** The PBMCs from two representative brucellosis patients (P1, P2) and a healthy blood donor control (NC) were stained by IFS with mAb 6C12. The IFS stained cells were observed by a Nikon Labophot photomicroscope under white or fluorescent light.

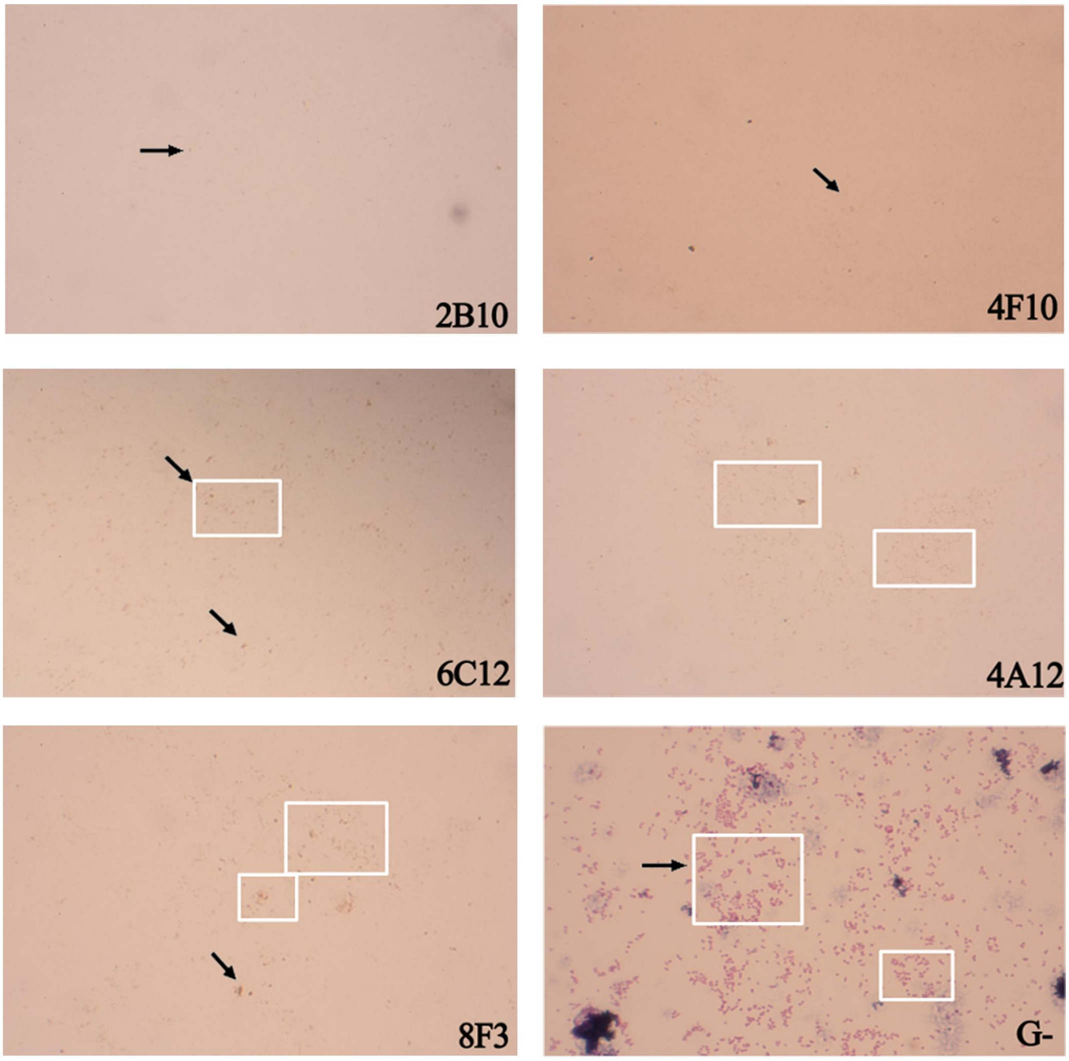

Figure S2. Identification of intact *B. melitensis* strain by ICS with mAbs. The intact bacteria of *B. melitensis* strain were stained by ICS with individual mAbs. G-, Gram staining for bacterial control of *B. melitensis* strain examined under white light with a microbiological microscope.

	1 **P2/mAb 4A12 & 4F10** 60
B. melitensis	MRTLKSLVIVSAALLPFSATAFAADAIQEQPPVPAPVEVAPQYSWAGGYTGLYLGYGWNK
Ochrobactrum anthropi	---------A---------------------------------M---------------
Rhizobiales 63-22	---------A----------------------A----Q----F---------------Q

	61 **P4/mAb 8F3** 120
B. melitensis	AKTSTVGSIKPDDWKAGAFAGWNFQQDQIVYGVEGDAGYSWAKKSKDGLEVKQGFEGSLR
Ochrobactrum anthropi	V--D---TV----M----Y---------F--------------T---------------
Rhizobiales 63-22	S-NEG--KSR--GM-LGGY-----E-------L------N----TR----T-------I-

	121 **P7/mAb 2B10** 180
B. melitensis	ARVGYDLNPVMPYLTAGIVGSQIKLNNGLDDESKFRVGWTAGAGLEAKLTDNILGRVEYR
Ochrobactrum anthropi	--L----------I---VA---V--D--F-------------------------------
Rhizobiales 63-22	G-L----------I---VA-------SAD-SA-----------M--R------------

	181 213
B. melitensis	YTQYGNKNYDLAGTTVRNKLDTQDFRVGIGYKF
Ochrobactrum anthropi	---F---T---GNES-------H-I---V----
Rhizobiales 63-22	---F-G-D-G-DDVS----YNSN-I---V----

Figure S3. Alignment of amino acid (aa) sequences of *B.melitensis* Omp25 with *Ochrobactrum anthropi* (NCBI reference sequence: WP_040129487.1) and *Rhizobiales* 63-22 (NCBI reference sequence: QJX99444.1) strains. The linear epitopes within *Brucella* Omp25

recognized by mAbs were indicated above the top sequence. Amino acids identified by one-letter code. Dashes indicate identity with the first aa sequence.
